# Report of the Distribution Committee

**Published:** 1920-12-18

**Authors:** John Tweedy

**Affiliations:** Chairman


**King Edward's Hospital Fund for London**—(*continued*)

The Statistical Report.

### REPORT OF THE DISTRIBUTION COMMITTEE.

(1) This is the third distribution made by the Fund this year. On June 22 the President and General Council decided to make an emergency distribution of £250,000 out of the accumulated funds in order to assist the London hospitals, having regard to their pressing maintenance needs; and the allocation of this sum amongst the hospitals was referred to the Distribution Committee with power to act. On July 23 the General Council, on the recommendation of the Distribution Committee, distributed another sum of £250,000, which had been generously entrusted to the Fund by the British Red Cross Society and the Order of St. John of Jerusalem for distribution on their behalf in aid of schemes of extension or improvement at hospitals able and willing to treat ex-Service men.

(2) The emergency distribution ordered by the Council on June 22 was completed by the Distribution Committee on July 5, when the grants set out in column 2 of the list appended to this Report were forwarded to the various hospitals. In the covering letters which accompanied the cheques it was stated that the object of these emergency grants was to assist the hospitals to carry on their work, and that they were not intended for the payment of debts already incurred, except where it appeared to the Hospital Committees that the circumstances were such that the reduction of debt was the best means of enabling the work to be carried on. The hospitals were also informed that the emergency grants would be taken into account at the ordinary distribution, and they were asked to consider in the meantime whether their finances could, without detriment, be improved by various methods—*e.g.*, patients' payments, organised contributions from employers and employees, the postponement of bed extensions, or, where the cost was high, greater economy in management.

(3) The amount placed at the disposal of the Distribution Committee for allocation amongst hospitals at the present ordinary distribution is £190,000. The amount distributed last year was £218,000, which was an increase of £28,000 on the previous year, and included an exceptional sum specially added to assist the hospitals to carry out renewals and repairs deferred on account of the war. The total

**King Edward's Hospital Fund for London**—(*continued*).

grants from the Fund, therefore, apart from the Red Cross grants, amount this year to £440,000, as against £218,000 in 1919 and £190,000 in 1918.

(4) The number of hospitals applying is 108.

(5) The distribution this year of over twice as much as last year is more than justified by the peculiar difficulties with which the hospitals are now faced. In 1919, for the first time for many years, the expenditure of the 108 hospitals on the books of the Fund, taken together, exceeded their income. The combined income, including legacies, amounted to £1,966,000, and the combined expenditure to £2,192,000, an aggregate excess of expenditure over income of £226,000. If the institutions whose income for the year exceeded their expenditure for the year are excluded, the aggregate excess of expenditure over income in 1919 was £341,000. This sudden change in the financial position of the London voluntary hospitals was due in the main to the great and unavoidable increase in the cost of every item of hospital expenditure; but this has unfortunately coincided with a serious falling-off in the subscribing power of their regular supporters. All the available evidence goes to show that these difficulties have rapidly increased since the end of 1919. The most urgent need in the present year has therefore been for substantial assistance to tide the hospitals over the immediate crisis, and to give them time to organise fresh sources of revenue. It was for this purpose that on July 5 the King's Fund made the emergency distribution of £250,000 out of its accumulated funds, in spite of the serious diminution in its annual income which would result therefrom.

(6) As foreshadowed by the Distribution Committee at the time, these emergency grants have been taken into account at the present ordinary distribution. It follows that, in comparing the grant to one hospital with that to another, or the grant in 1920 with the grant in 1919, the figure to be considered is the joint total of the ordinary grant and the emergency grant taken together.

(7) Since, however, the object of the emergency distribution was to assist the hospitals to tide over an immediate crisis, it was necessary for the Distribution Committee, in determining the amount of each grant, to give more weight than usual to the urgency of the pressing needs of each hospital, and less weight than usual to other considerations which are normally held to affect their relative claims on the Fund. The Committee felt obliged to apply this principle even to factors which, in some cases, might themselves be partially the cause of the comparative urgency of need, such as differences in economy of working, in the energy or skill devoted to raising funds for maintenance, or in the policy adopted on questions of hospital extensions and other forms of capital expenditure. As far as circumstances permitted, the Committee returned to their usual practice at the ordinary distribution.

(8) In view of the urgent need of maintenance grants, the Distribution Committee have kept the grants in aid of schemes of capital expenditure as low as was reasonably possible, having regard to the fact that numerous proposals for improvement or extension had already been deferred owing to the war, and were now long overdue. Many schemes of this kind, therefore, receive grants which on their merits alone would have been larger. In dealing with schemes which are necessary and urgent, the Distribution Committee have been greatly assisted by the generosity of the British Red Cross Society and the Order of St. John of Jerusalem in entrusting the King's Fund with the distribution on their behalf, on July 23 last, of £250,000 surplus funds for this purpose amongst hospitals able and willing to treat ex-Service men. In addition to the schemes at these hospitals, the Distribution Committee have had before them several equally urgent schemes at hospitals which were not eligible for Red Cross grants, such as hospitals for women or children. The total amount of the grants now recommended out of the ordinary distribution in aid of schemes of capital expenditure this year is £36,300, as against £43,375 in 1919. The total amount distributed by the Fund this year to hospitals may thus be subdivided as follows:—

| | £ | £ |
|---|---|---|
| Maintenance grants: | | |
| Emergency distribution, July 5 ... | 250,000 | |
| Ordinary distribution, December 14 | 153,700 | |
| Total maintenance grants ... | | 403,700 |
| Capital grants: | | |
| Surplus Red Cross Funds, July 23 ... | 250,000 | |
| Ordinary distribution, December 14 | 36,300 | |
| Total capital grants ... ... | | 286,300 |
| Total grants to hospitals ... | | £690,000 |

(9) No substantial capital grants have been made, either out of the Red Cross surplus funds or out of the ordinary distribution, except to schemes examined by the Distribution Committee after a detailed investigation by two of its members. Fifty-four schemes have been considered in this way during the current year, of which forty involved visits to the hospitals or to the sites. The Committee have continued to make a specially thorough examination of all schemes involving additional beds for in-patients. The number of beds proposed to be added has not materially changed since the Committee made a full report on the subject in connection with the Red Cross distribution on July 23.

(10) Twice this year the Distribution Committee have issued circulars calling attention to the standing request that schemes of capital expenditure might be submitted to the Fund before definite action was taken by the issue of an appeal or otherwise. In certain cases this has not been done, and capital grants which would otherwise have been made to the hospitals concerned have, in consequence, been reduced.

(11) The following grants in aid of schemes of building expenditure are recommended for renewal under the two years' rule:

General Lying-in Hospital, £1,000 in 1917 and £1,000 in 1918, to rebuilding on new site.

Great Northern Central Hospital, £1,000 in 1918 to new nurses' home.

London Lock Hospital, £1,500 in 1918 to reconstruction of the Harrow Road Hospital.

Queen Charlotte's Lying-in Hospital, £1,000 in 1913, £650 in 1917, and £750 in 1918, to new out-patient department.

Queen's Hospital for Children, £288 12s. 11d. (balance of £1,000) in 1917, and £1,250 in 1918, to improved out-patient accommodation.

St. Mary's Hospital for Women and Children, Plaistow, £1,500 in 1913, £1,000 in 1917, and £1,000 in 1918, to new nurses' home and out-patient department.

Victoria Hospital, £200 in 1916 to new heating apparatus.

(12) The Committee desire to record their appreciation of the generous help and ready assistance rendered by King George's Fund for Sailors in offering to provide the amount of the grant awarded to the Seamen's "Dreadnought" Hospital this year.

(13) The statistical report on the expenditure of the hospitals for the year 1919 shows a continued general rise in the cost of working. It also shows, at several hospitals, very large increases in cost per bed, due to decreases in the number of occupied beds consequent on the permanent closing of temporary beds for naval or military patients, or the temporary closing of permanent beds during the change over from military to civil use. Now that the war is over it has become necessary to consider whether the items omitted from the report during the past few years should be restored, and what changes are desirable and practicable in order to increase its usefulness in view of recent developments in the work of hospitals. The Distribution Committee welcome the appointment, by His Royal Highness the President, of the special Statistical Report Committee to advise on these questions.

(14) The Committee record with deep regret the death of Sir Henry Burdett, who had been closely associated with the Fund since its inception, and had been an active member of the Distribution Committee throughout. His wide knowledge of hospitals and deep interest in their work and in all that concerned the welfare of the sick poor were of the utmost value to the Committee, and he will be greatly missed.

(15) The Committee desire to express their thanks to Sir George Makins, who, in addition to his work as a member of the Executive Committee, very kindly consented to serve this year on the Distribution Committee in order to assist the medical members of the Committee with the examination of the numerous schemes submitted in connection with the Red Cross distribution. They also wish to thank the two Honorary Secretaries who are not on the Committee for similar help to the lay members.

(16) The Committee are again greatly indebted to the Visitors for their reports on the various hospitals, and have recommended that in some cases extracts from these reports should be forwarded to the Hospital Committees for their observations. Special inquiries have been made this year into the amount and condition of the accommodation provided for the domestic staff, on the same lines as the inquiry made a few years ago into the question of nurses' quarters.

(17) The list of grants is appended. The grant recommended out of the present ordinary distribution is set out in column 3. The grant made on July 5 out of the emergency distribution, and taken into account at the present distribution, is set out in column 2. The total amount granted this year (apart from any grant out of surplus Red Cross funds) is the sum-total of the figures in columns 2 and 3 taken together.

For the Committee,

JOHN TWEEDY,

*Chairman.*

November 30, 1920.

